# Analysis of upper airway CT-based radiomics in adult obstructive sleep apnea

**DOI:** 10.3389/fmed.2025.1737597

**Published:** 2026-01-13

**Authors:** Mingxuan Lin, Hui Qi, Kanghua Wang, Jie Wang, Haiyan Liu, Zhiying Nie, Yueqi Sun, Yunping Fan

**Affiliations:** Department of Otolaryngology, The Seventh Affiliated Hospital, Sun Yat-sen University, Shenzhen, Guangdong, China

**Keywords:** CT-based radiomics, machine learning, obstructive sleep apnea, severity prediction, upper airway

## Abstract

**Purpose:**

Computed tomography (CT) is a key tool for evaluating the upper airway in adult patients with obstructive sleep apnea (OSA). This study aimed to assess the value of CT-based radiomics for OSA evaluation.

**Methods:**

A total of 79 OSA patients and 19 healthy controls (HCs) were recruited between January 2023 and June 2024 and underwent upper airway CT scans. Radiomic features were extracted from CT images, and data processed using Python. Radiomic models were then developed to evaluate and predict OSA using ten machine learning algorithms. Model performance was assessed using area under the curve (AUC) values, calibration, and decision curve analysis (DCA).

**Results:**

The NaiveBayes machine learning algorithm based on radiomic features achieved the best result, and the AUCs for the Airway, Soft Tissue, and Entire in the test sets were 0.819, 0.812, and 0.854, respectively. In the test set, the Entire radiomic model was performed better than the other two models in OSA prediction with an AUC of 0.854 (95% CI, 0.674–1.000). The performance of Entire radiomic model was confirmed in the training and test set with satisfying predictive calibration and clinical application value.

**Conclusion:**

Upper airway CT-based radiomics model appears to be a promising tool. This radiomics-based method may be convenient and efficient for OSA assessment and prediction.

## 1 Introduction

Obstructive sleep apnea (OSA) is a prevalent chronic sleep disorder characterized by recurrent partial or complete upper airway collapse during sleep, leading to intermittent hypoxia, sleep fragmentation, hypercapnia, and sympathetic activation (1). It is estimated that nearly 1 billion adults worldwide and approximately 167 million in China alone, are affected by OSA, which substantially impairs quality of life and predisposes to cardiovascular disease, stroke, metabolic dysregulation, and elevated mortality (2–7). Therefore, early and accurate identification of OSA is essential to mitigate these serious complications.

Overnight polysomnography (PSG) remains the diagnostic gold standard, while home sleep apnea testing (HSAT) offers a convenient alternative for patients who are unable to access in-lab studies. However, both PSG and HSAT are hampered by high costs, limited availability, patient discomfort, and operational complexity, which constrain their utility as broad screening tools. Conventional radiological assessments, such as lateral cephalometry or soft-tissue imaging, can visualize upper airway anatomy but lack sensitivity for functional assessment and do not reliably quantify OSA severity (8).

Computed tomography (CT) of the upper airway provides high-resolution, three-dimensional evaluation of craniofacial structures, soft-tissue thickness, and airway volume, enabling precise localization of obstruction sites and morphological alterations (9). Yet, standard CT interpretation remains largely qualitative. Radiomics, a burgeoning field that converts medical images into mineable high-dimensional data, offers the potential to detect subvisual tissue heterogeneity by extracting quantitative features (e.g., texture, shape, and intensity) from defined regions of interest (10). Integrating these features with machine-learning algorithms and statistical models has yielded robust predictive tools across oncology, cardiology, and neurology (11–15), and can reveal microstructural changes in the upper airway that elude conventional imaging (16). To date, most studies applying machine learning to OSA prediction have relied on clinical and physiological parameters, or on radiographic metrics without harnessing full radiomic feature sets (17–22). The specific application of CT-based radiomic models to assess and predict OSA severity, and to correlate these imaging biomarkers with established clinical indices, remains underexplored.

In this study, we hypothesized that upper airway CT radiomics can provide an accurate and non-invasive means of stratifying OSA severity in adults. Therefore, we developed and validated radiomic signatures derived from segmented upper airway structures, integrated them with machine-learning classifiers, and evaluate their predictive performance against polysomnographic benchmarks. Our goal was to deliver a more efficient and accessible imaging-based tool to support early screening, diagnosis, and personalized management of OSA.

## 2 Method

### 2.1 Participant

Patients who visited the otorhinolaryngology clinic between January 2023 and June 2024 and complained of snoring or other symptoms of OSA were enrolled in our study. Healthy controls were recruited from our hospital.

All enrolled participants met the following inclusion criteria: (a) over 18 years old, (b) examined by an otolaryngologist, (c) underwent a CT scan, and (d) patients with OSA additionally underwent polysomnography.

The exclusion criteria were as follows: (a) incomplete clinical data, (b) severe diseases, (c) inadequate CT scan images, (d) craniofacial anomalies, (e) dental implants causing CT artifacts; and (f) an overnight sleep duration of less than 6 h.

OSA was defined according to the American and Chinese guidelines using the apnea-hypopnea index (AHI), with a threshold of >5 events/h (23, 24). A classification model was evaluated, trained and tested on the features extracted from patients with OSA and healthy controls. Additionally, clinical parameters included the lowest and average oxygen saturation (SaO_2_, percentage), body mass index (BMI, kg/m^2^).

**Figure 1:**
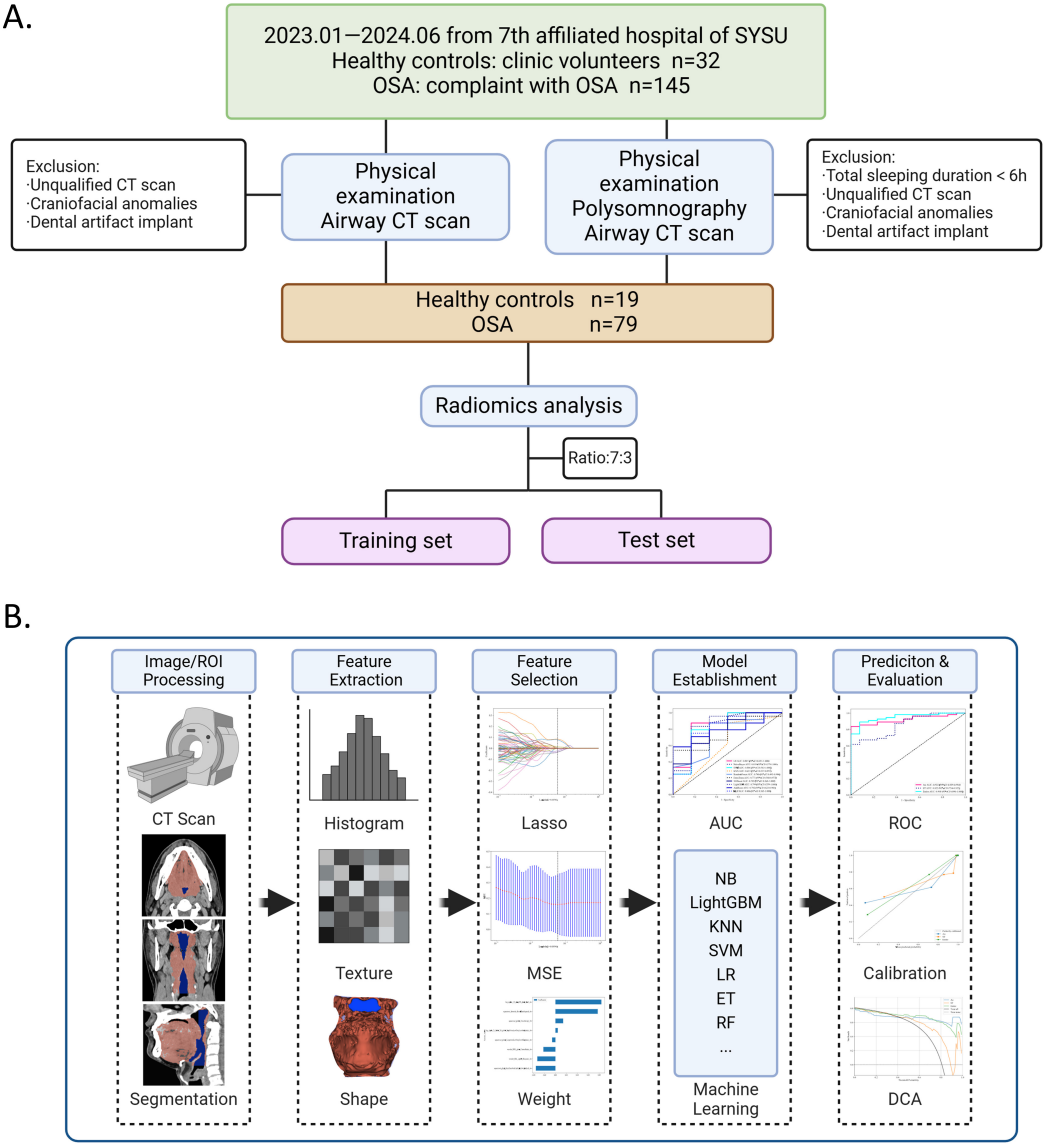
Flowchart of participant and radiomics in this study. **(A)** Participant enrollment flowchart shows the study progress for patients with OSA and healthy controls (HCs). **(B)** Radiomics flowchart, with image acquisition and processing (CT segmentation), radiomic feature extraction, and radiomic feature selection, establishment and evaluation of models, including Receiver operating characteristic ROCs of machine learning algorithms of feature models, and ROCs, calibration curve and DCA of radiomic models in different radiomic models.

The participant enrollment flowchart is presented in Figure 1A.

### 2.2 Image acquisition

All participants underwent an upper airway CT scan (128-row, Siemens, Germany) in the supine position. During scanning, the participants were instructed to keep their mouths closed, maintain occlusion of the maxillary and mandibular teeth, and breathe normally (eupnea), while refraining from speaking, swallowing, or moving their bodies. The scanning range was extended from the roof of the nasal cavity to the supraglottic region, using a tube voltage of 120 kV, a matrix size of 512 × 512, and a layer thickness of 0.625–1.5 mm.

### 2.3 Image segmentation

CT images were exported in Digital Imaging and Communications in Medicine (DICOM) format at a resolution of 300 dpi. The upper airway CT images were independently segmented by two experienced radiologists who were blinded to the patients' diagnoses. The regions of interest (ROIs) included the upper airway (AW), surrounding soft tissues (ST), and entire airway with soft tissues (EN). An experienced radiologist manually delineated the ROIs on each slice using open-source software 3D Slicer version 5.6.1 (https://www.slicer.org/).

### 2.4 Data preprocessing

The dataset was randomly divided into training and test sets in a 7:3 ratio. Cases in the training set were used to train the predictive model. Cases in the test set were used to independently assess the performance of the model. All images were resampled to a voxel size of 1 × 1 × 1 mm to standardize voxel spacing. An intraclass correlation coefficient (ICC) greater than 0.9 was considered robust.

### 2.5 Radiomics feature extraction

A total of 1,834 radiomic features from the ROI images were extracted using Pyradiomics' in-house feature analysis program (version 3.0.1). Radiomic features can be categorized into three groups: (i) geometry, (ii) intensity, and (iii) texture. Geometric features describe the three-dimensional shape characteristics of the ROIs. Intensity features describe the first-order statistical distribution of voxel intensities within regions of interest (ROIs). Texture features characterize the spatial patterns, including second- and higher-order distributions of voxel intensities. Texture features are extracted using various methods, including the gray-level co-occurrence matrix (GLCM), gray-level run-length matrix (GLRLM), gray-level size zone matrix (GLSZM), and neighborhood gray-tone difference matrix (NGTDM). To obtain high-throughput features, various methods were applied using filters (wavelet, Laplace-Gauss, square root, logarithm, exponential, gradient transform, and local binary mode transform). Z-score normalization was applied to address the issue of varying scales in the manual radiomic features.

To extract high-throughput features, our study applied several techniques: non-linear intensity transformations to image voxels (Square, SquareRoot, Logarithm, Gradient, LBP3D, and Exponential); Gaussian Laplace filtering with sigma values of 1, 2, and 3; and eight wavelet transform algorithms (LLL, LLH, LHL, LHH, HLL, HLH, HHL, and HHH) to compute first-order statistical and texture features.

The schematic diagram of the radiomics analysis workflow is presented in Figure 1B.

### 2.6 Radiomics feature selection

A Mann–Whitney U test was employed to screen for radiomic features. Only the radiomic features with a *p*-value < 0.05 were retained. An intraclass correlation coefficient (ICC) greater than 0.9 was considered robust. To ensure high repeatability, Spearman's rank correlation coefficient was computed between features; if the correlation coefficient between any two features exceeded 0.9, only one feature was retained. The minimum redundancy maximum relevance (mRMR) method was employed to select radiomic features. The least absolute shrinkage and selection operator (LASSO) regression model was applied to the discovery dataset for model construction. Depending on the regularization parameter λ, LASSO shrinks all regression coefficients toward zero and sets the coefficients of many irrelevant features exactly to zero. To determine an optimal λ, 10-fold cross-validation with a minimum error criterion was employed, whereby the final value of λ corresponded to the minimum cross-validation error (Supplementary Figure 1). The retained features with non-zero coefficients were used for regression model fitting and subsequently combined into the final model. The Python scikit-learn package was employed for LASSO regression modeling.

**Figure 2:**
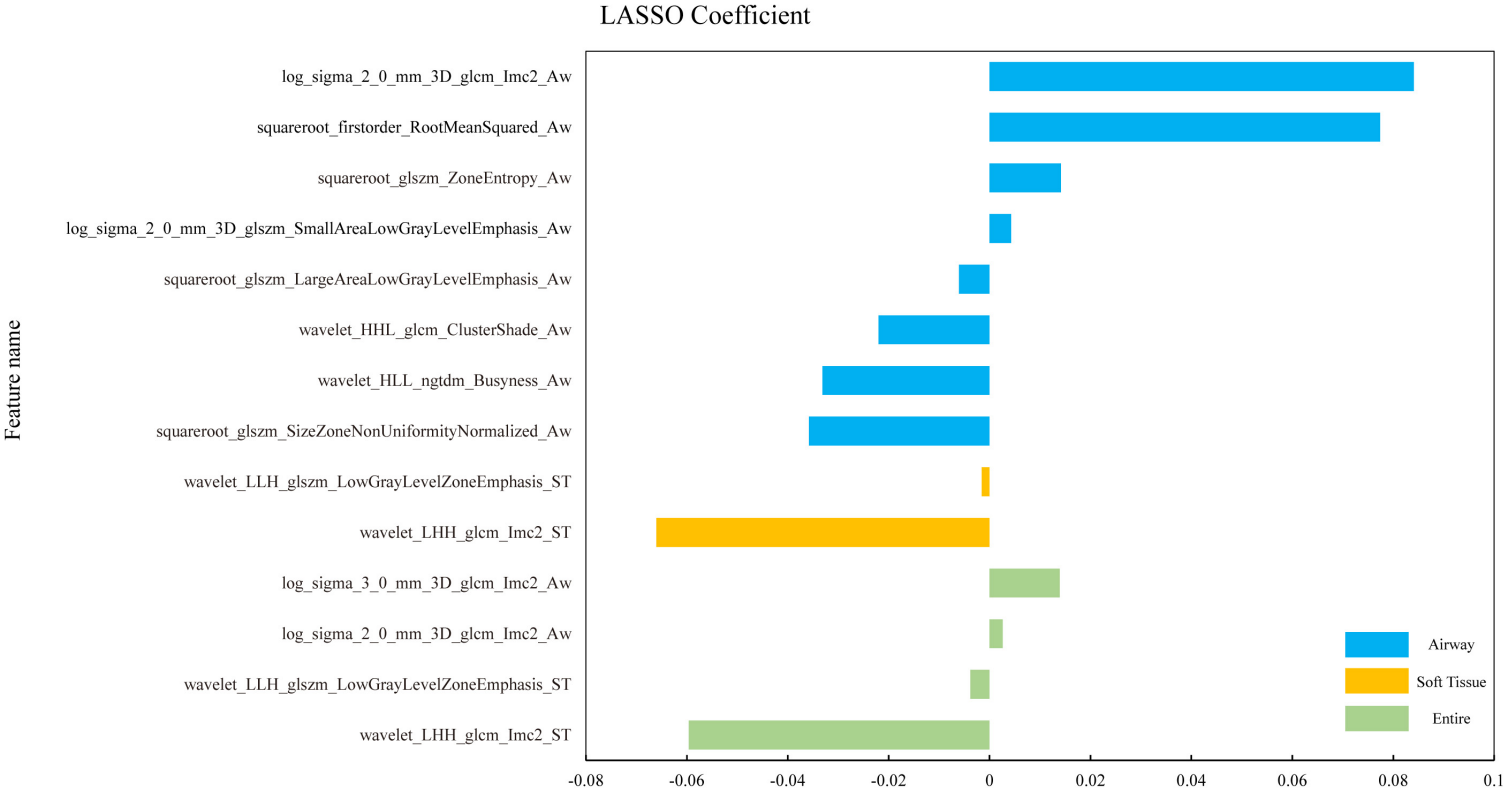
The histograms of coefficients of the final selected radiomic features. Coefficients of the final selected features from the three ROIs of OSA vs. HC in different colors. The horizontal coordinate represents the coefficient and the vertical coordinate represents the final selected features through the LASSO-algorithm.

The final results of feature extraction are presented in Figure 2.

### 2.7 Radiomics model establishment and analysis

To address class imbalance between OSA patients and healthy controls in the training set, the Synthetic Minority Over-sampling Technique (SMOTE) was applied within each training fold of the cross-validation to generate synthetic samples for the minority class by interpolating between nearest-neighbor instances, thereby reducing the bias toward the majority class and improving model learning. Importantly, SMOTE was performed only on the training data to avoid information leakage, while the corresponding validation fold was kept unchanged. The final retained radiomic features from the three ROIs were used to build machine learning models separately using ten algorithms: logistic regression (LR), Naïve Bayes (NB), support vector machine (SVM), k-nearest neighbors (KNN), random forest (RF), extra trees (ET), eXtreme Gradient Boost (XGBoost), Light Gradient Boosting Machine (LightGBM), Adaptive Boosting (AdaBoost), and multi-layer perceptron (MLP). These models were developed using 5-fold cross-validation. The performance was assessed through receiver operating characteristic (ROC) curves, along with additional evaluation metrics, including area under the curve (AUC), accuracy, sensitivity, specificity, positive predictive value (PPV), and negative predictive value (NPV).

Subsequently, the most appropriate machine learning models, which consistently demonstrated optimal performance across all three ROIs, were further analyzed. Finally, the discrimination among the three machine learning models was evaluated through ROC analysis with AUC values and DeLong's test. Decision curve analysis (DCA) and calibration curves were employed to evaluate the clinical utility of the candidate models and identify the optimal radiomic model for OSA evaluation.

### 2.8 Ethics

This study was approved by the Ethics Committee of the Seventh Affiliated Hospital of Sun Yat-sen University (KY-2025-385-01).

### 2.9 Statistical analysis

The Python statsmodels (version 0.13.2) package was used to perform statistical analysis. A *P*-value less than 0.05 was considered statistically significant. Continuous variables with a normal distribution were expressed as the mean ± standard deviation (SD) and analyzed using an independent samples *t*-test; continuous variables with a non-normal distribution were expressed as medians with the interquartile range [M (p25, p75)] and analyzed using the independent samples Mann-Whitney *U*-test; and categorical variables were expressed as frequencies (percentages) and analyzed using the χ^2^ test. The correlations between radiomic features and clinical parameters were analyzed using the Pearson correlation coefficient, with statistical significance set at *P* < 0.05.

## 3 Results

### 3.1 Participant characteristics

After screening, a total of 98 participants were finally enrolled in this study between January 2023 and June 2024. They were then randomly allocated to training and test sets. Participants were grouped as follows: healthy controls (*n* = 19), OSA (*n* = 79, AHI > 5 events/h), and 87 (88.8%) were male. The mean age of the HCs was 41.11 ± 14.26 years, their median BMI was 23.31 (22.56, 24.00) kg/m^2^. All OSA patients underwent PSG, their mean age was 38.41 ± 7.76, their mean BMI was 27.96 ± 3.47 kg/m^2^, the median AHI was 42.80 (23.30, 56.80) events/h, the median average SaO_2_ was 94.00 (91.00, 95.00) %, and the median lowest SaO_2_ was 75 (67.00, 82.00) %. Compared to healthy controls, patients with OSA had significantly higher weight and BMI in both the training and test sets.

The demographic and clinical parameters of the participants are summarized in Table 1.

**Table 1:** Summary of demographics and clinical parameters for the participants.

**Variables**	**Training (healthy control)**	**Training (OSA)**	***P* value**	**Test (healthy control)**	**Test (OSA)**	***P* value**
Gender			0.24			0.35
Female	3 (23.08)	4 (7.27)		2 (33.33)	2 (8.33)	
Male	10 (76.92)	51 (92.73)		4 (66.67)	22 (91.67)	
Age (years)	44.85 ± 15.50	37.75 ± 7.87	0.021^*^	34.50 (30.00,36.25)	38.00 (35.00,43.00)	0.046^*^
Height (m)	1.69 ± 0.08	1.71 ± 0.07	0.45	1.67 ± 0.08	1.71 ± 0.07	0.27
Weight (kg)	67.68 ± 10.62	81.8 ± 10.84	< 0.001^*^	63.62 ± 7.07	80.54 ± 12.02	0.03^*^
BMI (Kg/m^2^)	23.57 ± 2.18	28.10 ± 3.21	< 0.001^*^	22.67 ± 0.92	27.62 ± 4.07	0.007^*^
CT Scan Thickness (mm)	0.70 (0.50,1.00)	0.70 (0.70,0.70)	0.71	0.80 (0.50,1.31)	0.70 (0.70,0.70)	1.00
AHI (events/h)	-	46.1 (23.70,56.80)		-	41.30 (21.13,58.90)	
Avg SaO_2_ (%)	-	93.8 (91.00,95.00)		-	94.00 (91.25,95.00)	
Lowest SaO_2_ (%)	-	74.0 (64.00,81.00)		-	75.00 (70.00,83.75)	

Differences were considered statistically significant at *p* < 0.05(^*^). Continuous variables with normal distribution were expressed as mean ± SD ($\bar{x}\pm$s) and independent samples *t*-test was used; continuous variables with non-normal distribution were expressed as median (interquartile range) [M (p25, p75)] and independent samples Mann-Whitney *U*-test was used; and categorical variables were expressed as frequency (percentage) and χ2 test was used. OSA, obstructive sleep apnea; PSG, polysomnography; AHI, apnea-hypopnea index; BMI, body mass index (calculated as weight in kilograms divided by height in meters squared); HC, healthy control; Lowest SaO_2_, lowest arterial oxygen saturation; Avg SaO_2_, average arterial oxygen saturation; AW, airway; ST, soft tissue; EN, entire.

### 3.2 Feature selection and radiomics model establishment

**Figure 3:**
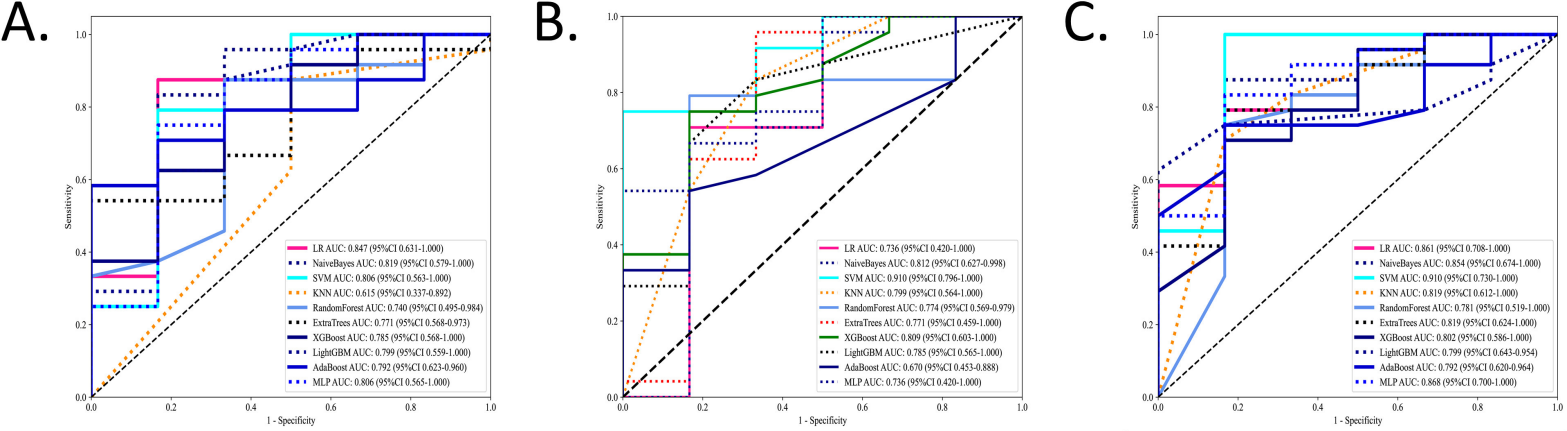
The performance of the machine learning models in the test sets. The ROCs of the ten different machine learning algorithms based on radiomic features for model establishment were compared in three ROIs in the test sets. Values of AUCs of ten machine learning algorithm models. **(A)** Airway. **(B)** Soft tissue. **(C)** Entire.

A total of 818 AW, 661 ST, and 1,479 EN features were extracted using the Mann–Whitney *U* test (*P* < 0.05). After ranking with the mRMR method, the top 40 significant radiomic features were selected. Using LASSO regression, the key features comprised eight AW features, two ST features, and four EN features. The features were subsequently input into ten different machine learning algorithms to build predictive models. ROC analysis was performed to compare the performance of the machine learning algorithms based on radiomic features for model establishment. The NB algorithms achieved the best AUCs in the test set; their performance was confirmed in all three radiomic models, with AUCs ranging from 0.812 to 0.854 (Figure 3).

The ROC analysis of the training sets is shown in Supplementary Figure 2. More details on ML algorithms are provided in Supplementary Tables 1–3.

### 3.3 Radiomic model performance for OSA prediction

**Figure 4:**
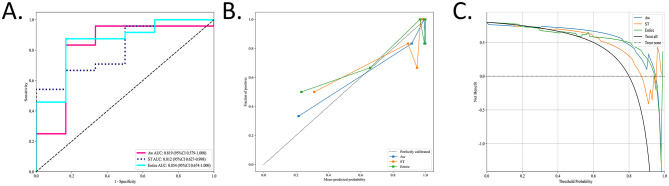
The performance of the radiomic models in the test sets. The performances of the three radiomic models for OSA prediction and evaluation were visually presented using ROC, calibration curve, and DCA. **(A)** Values of AUCs of three radiomic models. **(B)** Calibration curves of the three radiomic models. The diagonal gray dashed line indicates a perfect prediction, and the solid line indicate the performance of the models. When the solid line is close to the dotted line, the model works well. **(C)** DCAs of three different models. The gray line represents the assumption that “all patients are OSA,” and the dashed black line represents the assumption that “none are OSA.” The Entire model showed the highest net benefit compared with the other models in a large range of threshold probabilities, indicating the best clinical utility ability.

To identify the most accurate radiomic signature for OSA, three region-specific models (EN, AW, and ST) were evaluated using a naïve Bayes (NB) classifier in the independent test cohort. In this set, the EN model achieved the highest discrimination, with an area under the AUC of 0.854 (95% CI, 0.674–1.000), outperforming the AW model (AUC 0.819; 95% CI, 0.579–1.000), and the ST model (AUC 0.812; 95% CI, 0.627–0.998) (Figure 4A). DeLong's test confirmed that the AUC of EN model was significantly greater than those of both the AW and ST models (Table 2).

**Table 2:** Comparison of the difference in AUCs between various models using Delong's test.

**Group**	**Radiomic model**	**Training**	**Test**
		***P*** **-value**	
OSA vs. HC	EN vs. AW	0.001^*^	0.004^*^
	EN vs. ST	0.016^*^	0.035^*^
	AW vs. ST	0.254	0.959

Differences were considered statistically significant at *p* < 0.05 (^*^). AW, airway; ST, soft tissue; EN, entire.

Calibration curves for all three radiomic models closely approximated the 45° reference line, indicating excellent agreement between predicted probabilities and observed OSA status (Figure 4B). Decision curve analysis further demonstrated that each model provided a net benefit over the “treat-all” and “treat-none” strategies. Notably, the EN model delivered the greatest net benefit across a wide range of threshold probabilities (Figure 4C).

The corresponding AUCs, calibration curves, and DCA in the training set are presented in Supplementary Figure 3.

### 3.4 Correlation analysis between radiomics features and clinical parameters

The final selected radiomic features and clinical parameters (BMI, AHI, and lowest SaO_2_) were regarded as variables. Associations among all variables were analyzed using Pearson correlation coefficient (*P* < 0.05).

Some radiomic features exhibit moderate correlations with clinical parameters, suggesting that they may reflect certain pathological changes in OSA. In contrast, for features exhibiting no or low correlations with clinical parameters, they may capture unique information that is complementary to what is not captured by traditional clinical measures, or clinical and radiomic features might operate independently, capturing distinct dimensions of OSA.

**Figure 5:**
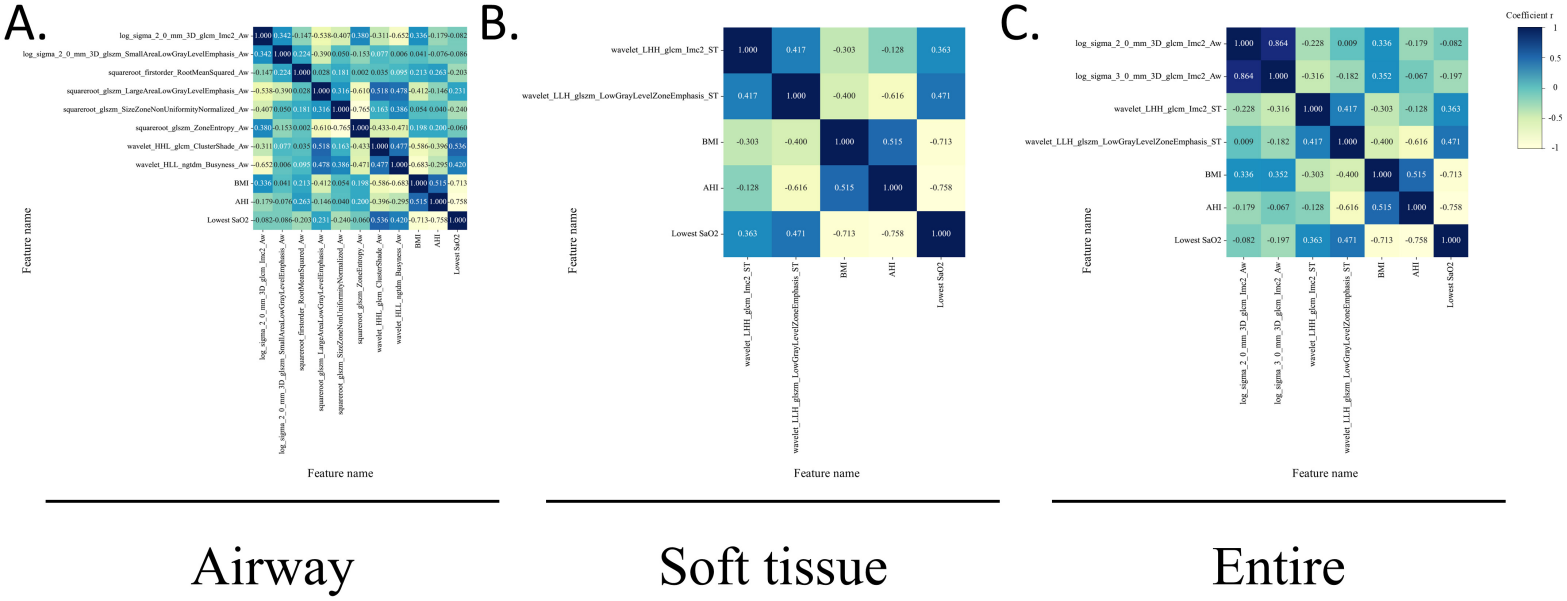
Correlations between radiomic features and clinical parameters in the test sets. The heatmap of the Pearson correlation coefficient between radiomics features and clinical parameters in the test sets, represented by different colors and absolute values. The radiomics features include the texture and first-order features, whereas clinical features included the BMI, AHI, and lowest SaO_2_. **(A)** Airway. **(B)** Soft tissue. **(C)** Entire.

The correlation analysis of the test and training sets in the radiomic models is shown in Figure 5 and Supplementary Figure 4.

## 4 Discussion

For the first time, our study demonstrated that the radiomics model may be used to evaluate and predict OSA. Radiomic features derived from upper airway CT scans were extracted using differential analysis and LASSO regression in 98 participants. The robustness of the AW, ST, and EN radiomic models was evaluated using machine learning algorithms. The comparative analysis showed that the EN model outperformed the other models in evaluating and predicting OSA. CT-based radiomic features may offer more details in OSA diagnosis, which emphasizes the value of 3D-reconstructed upper airway CT in OSA diagnosis. Given the widespread prevalence and severe complications of OSA, developing accessible and efficient methods for its evaluation and prediction is essential.

Obstructive sleep apnea (OSA) is a complex sleep disorder with potential risks of multi-systematic disease (25). Upper airway CT in the awake supine position is widely applied to investigate the pathogenesis of OSA (26, 27). Radiomics is an advanced image-based quantitative technique to promote ability to discover the subtle disease heterogeneity. The heterogeneity of OSA contributes to variations in grayscale, shape, and texture in imaging, which can be analyzed using radiomics. To the best of our knowledge, our study is the first time to define ROIs encompassing the entire airway cavity, including the airway lumen and soft tissue, to maximize disease information extraction. We developed a radiomics model consisting of key radiomic features to identify OSA using 10 different machine learning algorithms. Radiomic features were categorized into two types, each offering potentially interpretable information: (a) first-order features (RootMeanSquared), which display the grayscale values of ROI and thus may indicate uneven distribution; (b) texture features (GLCM, GLSZM, NGTDM, etc.), that quantify texture complexity, potentially indicating tissue remodeling (e.g., fibrosis, adipose deposition) or gas density changes in the airway lumen. To minimize bias, bone structures and dental artifact implants were excluded during threshold segmentation to ensure the validity of the CT radiomics models.

In our study, the NaiveBayes algorithm performed the best in the training sets, achieving AUCs from 0.855 to 0.948, which was confirmed in the test sets (AUCs, ranging from 0.812 to 0.854). Subsequently, the potentials of the AW, ST, and EN radiomic models were compared to assess and predict OSA. The EN model outperformed the other models in the test set (AUC 0.854, 95% CI, 0.674–1.000). Positive predictive value (PPV), and negative predictive value (NPV) by comparing the accuracy, sensitivity, specificity, Delong's test, DCA, calibration curve, the performance of the EN model was found to be optimal for the AW and ST model. Our study also found that radiomic features were associated with texture and first-order features in upper airway CT images, which form the basis of the OSA prediction model. The fine structure and heterogeneity of tissues can be quantified using texture features (28). These features reflect the heterogeneity and complexity of airway and peri-airway soft tissues. Gray scale fluctuation in the airway region indicates uneven gas distribution, while distribution in soft tissue regions reflects the degree of adipose deposition or fibrosis. Chronic intermittent hypoxia-induced inflammation contributes to tissue damage (29). Patients with severe OSA often show increased texture feature complexity in soft tissues, likely due to mixed adipose and fibrous tissue distribution (30). Soft tissue hyperplasia in the upper airway is commonly observed in OSA patients, with increased volumes of the lateral pharyngeal wall and soft tissues being significant contributors to OSA severity (31). In obese patients with OSA, soft tissue areas often exhibit a higher proportion of low-density adipose tissue (32). Tissue remodeling can reduce the cross-sectional area of the pharyngeal lumen, leading to upper airway obstruction during sleep (33). Peri-airway soft tissues play a critical role in maintaining airway patency (1). Changes in lumen morphology and structure directly affect airflow patency and ultimately result in increased pharyngeal critical closing pressure (Pcrit) (34). Altered tissue mechanics and collapsibility could lead to upper airway narrowing, furthermore to uneven gas distribution within the lumen (35). Uneven gas distribution was also a common phenomenon in obstructive respiratory diseases, CT-based quantification usually relied on gas density threshold metrics to evaluate its severities (36–38). Previous investigations found that computational fluid dynamics (CFD) based on upper airway CT scans can evaluate heterogeneous alterations in airflow distribution due to varying degrees of airway collapse or obstruction among OSA patients (39). Aerodynamic changes caused by a narrowed lumen can alter gas distribution and flow velocity (40), manifesting as grayscale changes and imbalanced distributions. Therefore, this non-uniform intraluminal aeration and airflow patterns may increase voxel-level heterogeneity within the lumen segmentation and thereby shift both first-order intensity features and texture features. Thus, upper airway collapse during sleep can exacerbate airflow fluctuations, reinforcing uneven gas distribution and radiomic heterogeneity. Upper airway structure or function, disrupt the balance between respiratory drive and load compensation, and exacerbate OSA events (41). Notably, we discovered correlations between radiomic features and clinical parameters. The moderate correlation indicates that radiomic features hold certain biological significance in revealing disease severity. Features exhibiting no or low correlations may offer additional, non-redundant pathological information that can contribute to the construction of more comprehensive risk assessment models. These results suggest that upper airway CT-based radiomics has the potential to assess and predict OSA.

Thus, direct and efficient diagnosis and assessment of OSA remains a clinical challenge. Radiomics has shown potential for evaluating and predicting OSA by enhancing the detection of subtle disease transformations and identifying lesions in normal-appearing tissues (42). Our study demonstrated that upper airway radiomics, when combined with machine learning models, can be effectively applied to clinical prediction tasks (43). Our findings regarding texture features extracted from the airways of OSA patients suggest a relationship with uneven gas distribution caused by airway narrowing, consisting with previous studies on airway aerodynamics in OSA (39). Recent studies have developed upper airway models using AI to predict OSA severity, but their limited delineation of regions may hinder comprehensive assessments (22, 44). Previous studies have tried various indicators to predict OSA severity (20, 44–48), these approaches may lack efficiency and robustness for clinical application. Compared to other prediction methods, our study demonstrated that radiomics models objectively quantify upper airway and soft tissue features, providing a non-invasive, reliable, cost-effective, and accessible method for assessing and predicting OSA. Physicians can integrate upper airway CT in conjunction with routine physical examination to rapidly diagnose potential OSA patients.

This study had several limitations. First, this was a single-center study with a small sample size, so future studies with larger and more sex-balanced samples are warranted to validate and refine the proposed model and to explore potential sex-specific differences in radiomic characteristics and model performance. Second, an external validation set is required to develop a more comprehensive model and to enhance generalizability. Finally, the manual delineation of ROIs is time-consuming; therefore, automatic segmentation should be explored to minimize artificial errors in the future. Additionally, more precise segmentation approaches should be considered to improve the predictive outcomes.

## 5 Conclusion

Despite these limitations, this study, for the first time, proposes that upper airway CT-based radiomics shows the potential to assess and predict OSA and its severity. The radiomic model demonstrated a satisfactory performance. This radiomics study may provide a new, low-cost, and efficient pathway for OSA diagnosis and severity assessment.

## Data Availability

The raw data supporting the conclusions of this article will be made available by the authors, without undue reservation.
