# Supplementary material for: Analysis of upper airway CT-based radiomics in adult obstructive sleep apnea
Source: Front Med (Lausanne). 2026 Jan 13;12:1737597. doi: 10.3389/fmed.2025.1737597 (PMC12835338; doi:10.3389/fmed.2025.1737597)
Supplement: Supplementary file 1 [file Table_1.docx]

| Algorithm | Task | AUC (95%CI) | Accuracy | Sensitivity | Specificity | Recall | F1 |
| --- | --- | --- | --- | --- | --- | --- | --- |
| LR | train | 0.966(0.9295-1.0000) | 0.868 | 0.836 | 1.000 | 0.836 | 0.911 |
|  | test | 0.847(0.6309-1.0000) | 0.833 | 0.833 | 0.833 | 0.833 | 0.889 |
| NB | train | 0.922(0.8590-0.9843) | 0.853 | 0.818 | 1.000 | 0.818 | 0.900 |
|  | test | 0.819(0.5787-1.0000) | 0.800 | 0.792 | 0.833 | 0.792 | 0.864 |
| SVM | train | 0.993(0.9805-1.0000) | 0.941 | 0.927 | 1.000 | 0.927 | 0.962 |
|  | test | 0.806(0.5627-1.0000) | 0.767 | 0.750 | 0.833 | 0.750 | 0.837 |
| KNN | train | 0.946(0.8968-0.9955) | 0.191 | 0.000 | 1.000 | 0.000 | NaN |
|  | test | 0.615(0.3370-0.8922) | 0.600 | 0.625 | 0.500 | 0.625 | 0.714 |
| RF | train | 0.987(0.9658-1.0000) | 0.941 | 0.945 | 0.923 | 0.945 | 0.963 |
|  | test | 0.740(0.4949-0.9842) | 0.800 | 0.833 | 0.667 | 0.833 | 0.870 |
| ET | train | 0.965(0.9207-1.0000) | 0.897 | 0.891 | 0.923 | 0.891 | 0.933 |
|  | test | 0.771(0.5683-0.9734) | 0.600 | 0.500 | 1.000 | 0.500 | 0.667 |
| XGBoost | train | 1.000(1.0000-1.0000) | 0.985 | 0.982 | 1.000 | 0.982 | 0.991 |
|  | test | 0.785(0.5684-1.0000) | 0.633 | 0.583 | 0.833 | 0.583 | 0.718 |
| LightGBM | train | 0.931(0.8734-0.9881) | 0.853 | 0.818 | 1.000 | 0.818 | 0.900 |
|  | test | 0.799(0.5593-1.0000) | 0.800 | 0.833 | 0.667 | 0.833 | 0.870 |
| AdaBoost | train | 1.000(1.0000-1.0000) | 0.985 | 0.982 | 1.000 | 0.982 | 0.991 |
|  | test | 0.792(0.6230-0.9604) | 0.633 | 0.542 | 1.000 | 0.542 | 0.703 |
| MLP | train | 0.966(0.9289-1.0000) | 0.912 | 0.909 | 0.923 | 0.909 | 0.943 |
|  | test | 0.806(0.5645-1.0000) | 0.733 | 0.708 | 0.833 | 0.708 | 0.810 |

**Table S1 Machine learning model performance AW region**

**Table S2 Model performance of radiomics models in ST region**

| Algorithm | Task | AUC (95%CI) | Accuracy | Sensitivity | Specificity | Recall | F1 |
| --- | --- | --- | --- | --- | --- | --- | --- |
| LR | train | 0.860(0.7629-0.9573) | 0.676 | 0.600 | 1.000 | 0.600 | 0.750 |
|  | test | 0.736(0.4196-1.0000) | 0.700 | 0.667 | 0.833 | 0.667 | 0.780 |
| NB | train | 0.855(0.7543-0.9548) | 0.676 | 0.600 | 1.000 | 0.600 | 0.750 |
|  | test | 0.812(0.6274-0.9976) | 0.600 | 0.500 | 1.000 | 0.500 | 0.667 |
| SVM | train | 0.876(0.7808-0.9703) | 0.824 | 0.836 | 0.769 | 0.836 | 0.885 |
|  | test | 0.910(0.7965-1.0000) | 0.767 | 0.708 | 1.000 | 0.708 | 0.829 |
| KNN | train | 0.891(0.8153-0.9666) | 0.191 | 0.000 | 1.000 | 0.000 | NaN |
|  | test | 0.799(0.5640-1.0000) | 0.600 | 0.542 | 0.833 | 0.542 | 0.684 |
| RF | train | 0.958(0.9127-1.0000) | 0.868 | 0.855 | 0.923 | 0.855 | 0.913 |
|  | test | 0.774(0.5693-0.9793) | 0.767 | 0.750 | 0.833 | 0.750 | 0.837 |
| ET | train | 0.893(0.8079-0.9781) | 0.809 | 0.818 | 0.769 | 0.818 | 0.874 |
|  | test | 0.771(0.4588-1.0000) | 0.867 | 0.917 | 0.667 | 0.917 | 0.917 |
| XGBoost | train | 0.990(0.9724-1.0000) | 0.926 | 0.909 | 1.000 | 0.909 | 0.952 |
|  | test | 0.809(0.6027-1.0000) | 0.700 | 0.667 | 0.833 | 0.667 | 0.780 |
| LightGBM | train | 0.846(0.7646-0.9278) | 0.632 | 0.545 | 1.000 | 0.545 | 0.706 |
|  | test | 0.785(0.5649-1.0000) | 0.600 | 0.542 | 0.833 | 0.542 | 0.684 |
| AdaBoost | train | 0.983(0.9594-1.0000) | 0.824 | 0.782 | 1.000 | 0.782 | 0.878 |
|  | test | 0.670(0.4527-0.8876) | 0.467 | 0.375 | 0.833 | 0.375 | 0.529 |
| MLP | train | 0.859(0.7610-0.9565) | 0.706 | 0.636 | 1.000 | 0.636 | 0.778 |
|  | test | 0.736(0.4195-1.0000) | 0.667 | 0.625 | 0.833 | 0.625 | 0.750 |

**Table S3 Model performance of radiomics models in EN region**

| Algorithm | Task | AUC (95%CI) | Accuracy | Sensitivity | Specificity | Recall | F1 |
| --- | --- | --- | --- | --- | --- | --- | --- |
| LR | train | 0.944(0.8914-0.9967) | 0.868 | 0.855 | 0.923 | 0.979 | 0.855 |
|  | test | 0.861(0.7076-1.0000) | 0.767 | 0.750 | 0.833 | 0.947 | 0.750 |
| NB | train | 0.948(0.8961-1.0000) | 0.882 | 0.873 | 0.923 | 0.980 | 0.873 |
|  | test | 0.854(0.6741-1.0000) | 0.833 | 0.833 | 0.833 | 0.952 | 0.833 |
| SVM | train | 0.919(0.8009-1.0000) | 0.897 | 0.891 | 0.923 | 0.980 | 0.891 |
|  | test | 0.910(0.7296-1.0000) | 0.933 | 0.958 | 0.833 | 0.958 | 0.958 |
| KNN | train | 0.960(0.9204-0.9999) | 0.809 | 0.764 | 1.000 | 1.000 | 0.764 |
|  | test | 0.819(0.6124-1.0000) | 0.200 | 0.000 | 1.000 | 0.000 | 0.000 |
| RF | train | 0.985(0.9612-1.0000) | 0.882 | 0.855 | 1.000 | 1.000 | 0.855 |
|  | test | 0.781(0.5195-1.0000) | 0.733 | 0.708 | 0.833 | 0.944 | 0.708 |
| ET | train | 0.969(0.9265-1.0000) | 0.941 | 0.945 | 0.923 | 0.981 | 0.945 |
|  | test | 0.819(0.6239-1.0000) | 0.767 | 0.750 | 0.833 | 0.947 | 0.750 |
| XGBoost | train | 0.992(0.9771-1.0000) | 0.912 | 0.891 | 1.000 | 1.000 | 0.891 |
|  | test | 0.802(0.5863-1.0000) | 0.700 | 0.667 | 0.833 | 0.941 | 0.667 |
| LightGBM | train | 0.890(0.8198-0.9593) | 0.750 | 0.691 | 1.000 | 1.000 | 0.691 |
|  | test | 0.799(0.6429-0.9543) | 0.667 | 0.583 | 1.000 | 1.000 | 0.583 |
| AdaBoost | train | 0.993(0.9808-1.0000) | 0.926 | 0.909 | 1.000 | 1.000 | 0.909 |
|  | test | 0.792(0.6197-0.9636) | 0.733 | 0.708 | 0.833 | 0.944 | 0.708 |
| MLP | train | 0.955(0.9084-1.0000) | 0.897 | 0.891 | 0.923 | 0.980 | 0.891 |
|  | test | 0.868(0.6998-1.0000) | 0.800 | 0.792 | 0.833 | 0.950 | 0.792 |

**Supplementary Figure legends**

**Supplementary Figure 1 Radiomic feature selection based on LASSO algorithm.**

The radiomic features were further selected based on LASSO algorithm.

(A-F) Ten-fold cross-validated coefficients and MSE of OSA vs. HC.

(A, B) Airway; (C, D) Soft tissue; (E, F) Entire.

**Supplementary Figure 2 The performance of the machine learning models in the training sets.**

The ROCs of the ten different machine learning algorithms based on radiomic features for model establishment were compared in three ROIs in the training sets. The values of AUCs of ten machine learning algorithms models.

(A) Airway. (B) Soft tissue; (C) Entire.

**Supplementary Figure 3 The performance of the radiomic models in the training sets.**

The performances of three radiomic models for OSA prediction and evaluation were visually presented by ROC, calibration curve, and DCA.

(A) The values of AUCs of three radiomic models;

(B) Calibration curves of the three radiomic models. The diagonal gray dashed line indicates a perfect prediction, and the solid line indicate the performance of the models. When the solid line is close to the dotted line, the model works well;

(C) The DCAs of the three models in different models. The gray line represents the assumption that “all patients are OSA”, and the dashed black line represents the assumption that “none are OSA”. The Entire model shows the highest net benefit compared with the other models in a large range of threshold probability, indicating the best clinical utility ability.

**Supplementary Figure 4 Correlations between radiomic features and clinical parameters in the training sets.**

The heatmap of Pearson correlation coefficient between radiomics features and clinical parameters in the training sets, represented by different colors and absolute values. The radiomics features include the texture and first-order features, whereas clinical features included the BMI, AHI, and lowest SaO_2_.

(A) Airway. (B) Soft tissue; (C) Entire.
