# Supplementary figures and images for: Analysis of upper airway CT-based radiomics in adult obstructive sleep apnea
Source: Front Med (Lausanne). 2026 Jan 13;12:1737597. doi: 10.3389/fmed.2025.1737597 (PMC12835338; doi:10.3389/fmed.2025.1737597)

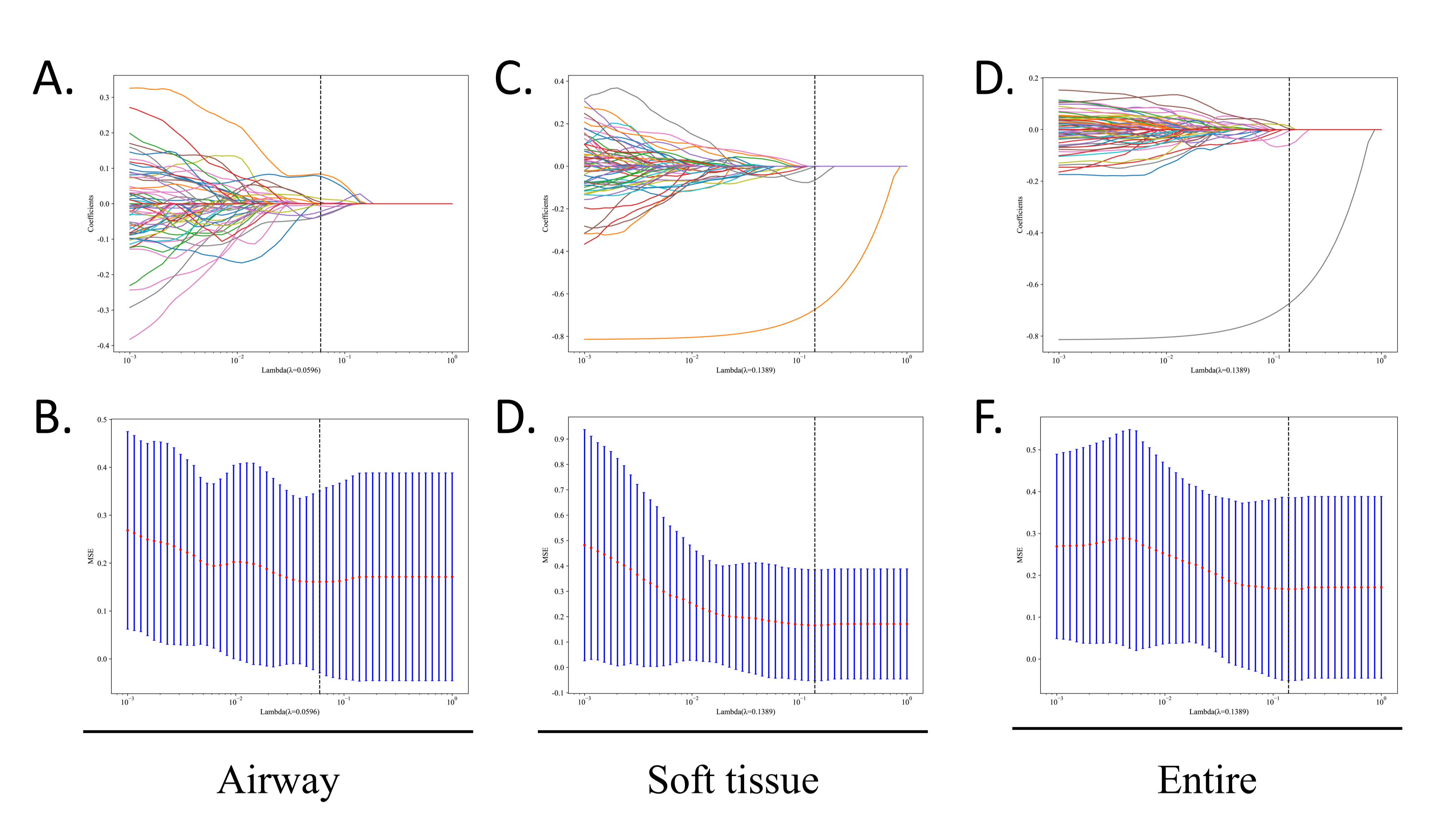

Supplement: Supplementary file 2 [file Image_1.tif]

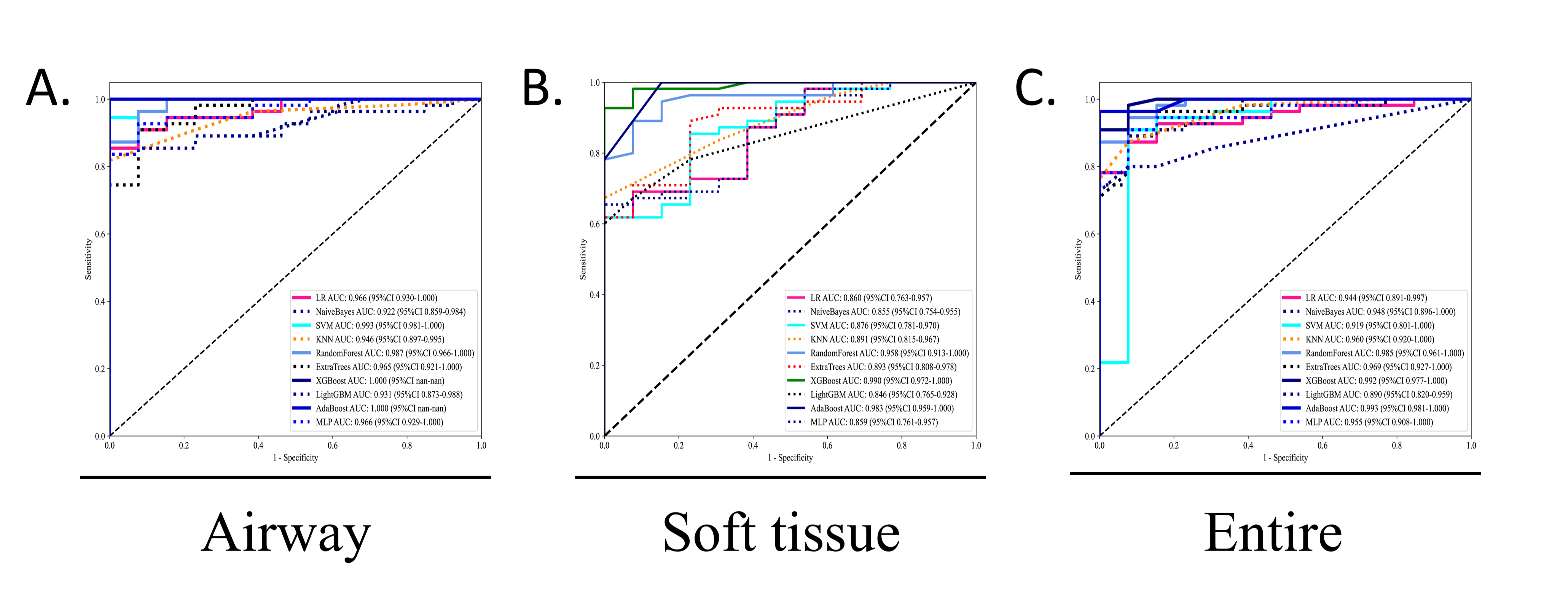

Supplement: Supplementary file 3 [file Image_2.tif]

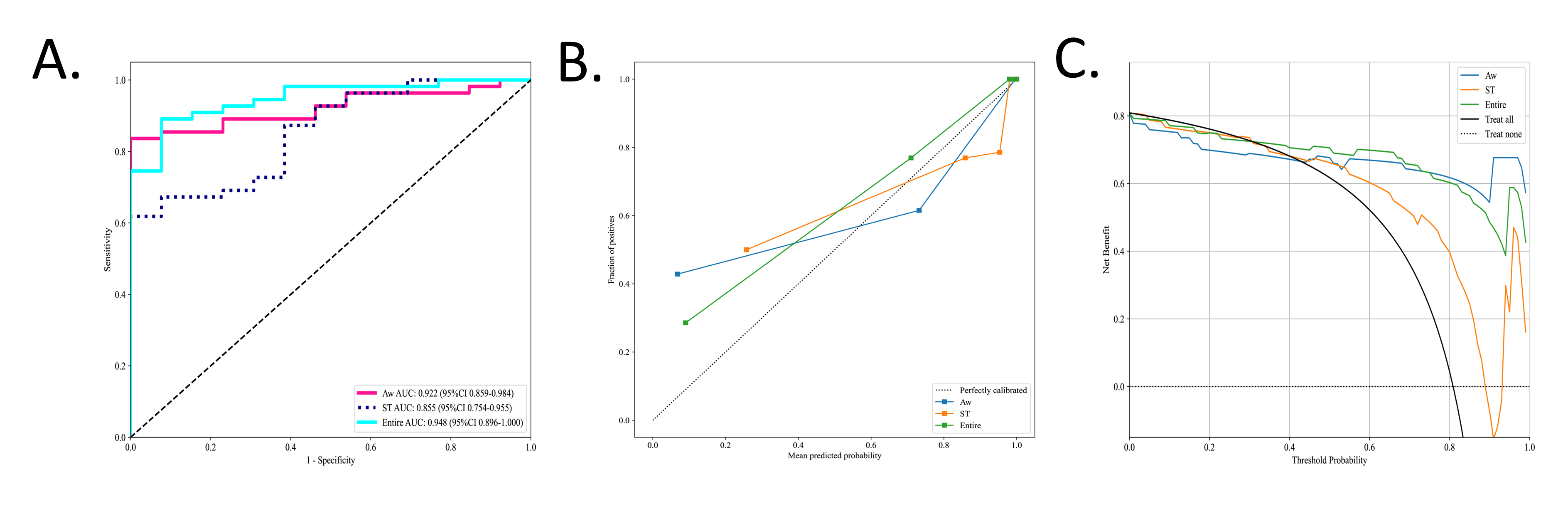

Supplement: Supplementary file 4 [file Image_3.tif]

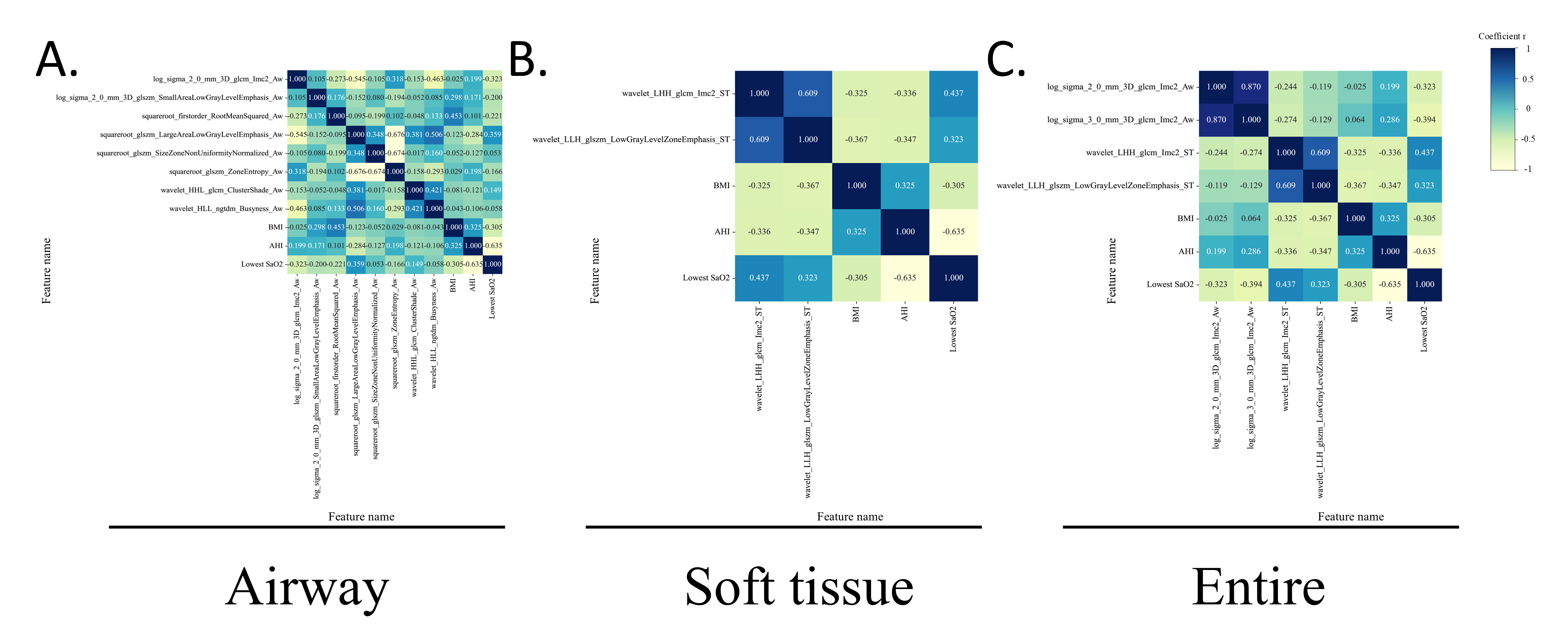

Supplement: Supplementary file 5 [file Image_4.tif]
